# Obituary: Professor Arun Kumar Gupta

**Published:** 2008

**Authors:** Surya Bhan

**Affiliations:** *New Delhi, India. E-mail: suryabhan@hotmail.com*


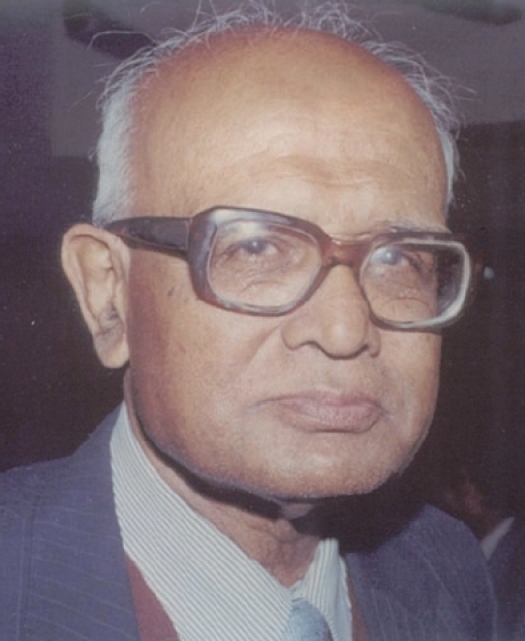


**Prof. A. K. Gupta, M.S, F.R.C.S, M.Ch.Orth 1917-2007**

Professor Arun Kumar Gupta, the doyen of Indian Orthopaedics, died on 30^th^ December 2007, just after his 90^th^ birthday. He fought an extended battle against cancer in the last two years of his life. Throughout his active life, he displayed admirable qualities of responsibility and commitment to both patient care and teaching.

Prof. A. K. Gupta was born on 17^th^ December 1917. He entered Patna Medical College and obtained M.B.B.S. (1941) and M.S. General Surgery (1944). He then joined the Indian Army and served in India, Singapore and Malaysia. He left the Short Service Commission of the Army in 1947 and went to the UK, where he worked at United Liverpool Hospital. He obtained M.Ch. (Orth) from Liverpool (1951) and F.R.C.S. (1952) from Royal College of Surgeons, England. He was full of national pride and so returned to India in 1953 to join Irwin Hospital (now Maulana Azad Medical College and LNJPN Hospital) in Delhi where he remained till 1955. His next appointment was as Lecturer of Orthopaedics at S.N. Medical College, Agra from 1955 to 1957. He then moved to G.S.V.M. Medical College, Kanpur, as Reader in Orthopaedics and was responsible for establishing the Department of Orthopaedics there. He became Professor and Head of the Department in 1959, a position he held till his retirement in 1976. The course for M.S.Orth. degree was started at Kanpur in 1960 and in the next 15 years till his retirement, 38 M.S. and 36 D.Orth surgeons were trained. Most of his students achieved senior professional and academic positions in India and overseas.

Prof. A. K. Gupta had a positive and optimistic attitude, and did not believe in wasting time and complaining about problems. He never appeared rushed and no one ever heard him speak in a harsh voice to anyone around him. He had appropriate facial expressions, body movements and a friendly attitude, which are ingredients of lasting relationship. He was a dedicated teacher, tireless surgeon and a respected leader. Educating orthopaedic residents never took a backseat to anything. Prof. Gupta was a lifelong scholar. He read voraciously and had broad depth of knowledge of orthopaedics as well as of literature and current events.

Prof. A. K. Gupta had wide-ranging clinical interests, but the topics very close to his heart were rickets and osteomalacia, paediatric femoral neck fractures, osteotomies around hip, correction of foot deformities and hand and lower limb deformities of poliomyelitis, and Hansen's disease.

He played an active role in Indian Orthopaedic Association and was its Secretary from 1963 to 1966 and then held the chair of vice-president in 1967. He was honoured with Presidency of Indian Orthopaedic Association (IOA) in 1975-1976 and was awarded the highest honour of “IOA - Lifetime Achievement Award” at the 51^st^ Annual Conference of Indian Orthopaedic Association in 2006 at Delhi. “A.K. Gupta Trophy” is awarded to the best chapter of IOA, and the best paper presented at UP Chapter of Indian Orthopaedic Association (UPCON), gets “A.K. Gupta Gold Medal”. UPCON - 2008 and the January 2008 issue of Bone and Joint Diseases were dedicated to the memory of Prof. A. K. Gupta.

Prof. A. K. Gupta spent a lot of time trying to impart compassion as a surgical skill. He was a very loving man and will be missed dearly not only by his family, but also by his students and all who ever came in contact with him.

